# Extracellular Vesicle-Encapsulated miR-29b-3p Released From Bone Marrow-Derived Mesenchymal Stem Cells Underpins Osteogenic Differentiation

**DOI:** 10.3389/fcell.2020.581545

**Published:** 2021-01-22

**Authors:** Xueliang Zhang, Wenji Wang, Yongping Wang, Haiyan Zhao, Xingwen Han, Tong Zhao, Peng Qu

**Affiliations:** Department of Orthopaedics, The First Hospital of Lanzhou University, Lanzhou, China

**Keywords:** microRNA-29b-3p, KDM5A, SOCS1, NF-κB, extracellular vesicles, osteogenic differentiation

## Abstract

**Objective:**

Mesenchymal stem cells (MSCs) confer therapeutic benefits in various pathologies and cancers by releasing extracellular vesicles (EVs) loaded with bioactive compounds. Herein, we identified bone marrow MSC (BMSC)-derived EVs harboring microRNA (miR)-29b-3p to regulate osteogenic differentiation through effects on the suppressor of cytokine signaling 1 (SOCS1)/nuclear factor (NF)-κB pathway *via* targeting of lysine demethylase 5A (KDM5A) in osteoporosis.

**Methods:**

We quantified the miR-29b-3p in BMSC-derived EVs from bone marrow specimens of osteoporotic patients and non-osteoporotic patients during total hip arthroplasty (THA). miR-29b-3p targeting KDM5A was confirmed by promoter luciferase assay, and enrichment of KDM5A in the promoter region of SOCS1 was analyzed by chromatin immunoprecipitation (ChIP). The expression and translocation of NF-κB to the nucleus were detected by western blot analysis and immunofluorescence staining, respectively. An ovariectomized (OVX) osteoporosis mouse model was established to further confirm the *in vitro* findings.

**Results:**

BMSC-derived EVs of osteoporotic patients exhibited downregulated miR-29b-3p. EV-encapsulated miR-29b-3p from BMSCs potentiated osteogenic differentiation by specifically inhibiting KDM5A. KDM5A inhibited osteogenic differentiation by the regulation of H3K4me3 and H3K27ac of SOCS1. SOCS1 potentiated osteogenic differentiation by inhibiting NF-κB pathway.

**Conclusion:**

EV-encapsulated miR-29b-3p derived from BMSCs potentiated osteogenic differentiation through blockade of the SOCS1/NF-κB pathway by inhibition of KDM5A.

## Introduction

Osteoporosis is one of the most prevailing debilitating skeletal disorders especially in the elderly population characterized by bone mineral density (BMD) reduction and bone structure destruction (Ensrud and Crandall, 2017). Osteoporotic fractures can cause acute and chronic pain that mainly affect elderly patients with multiple comorbidities (Vellucci et al., 2018). In the context of osteoporosis, there is increased adipogenesis occurring in conjunction with reduced bone formation (Pino et al., 2012). If impaired osteogenesis of adult mesenchymal stem cells (MSCs) constitutes a causative mechanism of age-related osteoporosis, this would imply that restoring the osteogenic differentiation of bone marrow-derived MSCs (BMSCs) could be a potential therapeutic strategy for osteoporosis (Tan et al., 2015; Fang et al., 2019). MSCs achieve their therapeutic effects *in vivo via* a paracrine action. Extracellular vesicles (EVs) are well-established as a key paracrine-acting factor released by MSCs that contribute to their therapeutic potency in various disease models (Alcaraz et al., 2019). For example, EVs derived from stem cells prevented bone loss in a murine osteoporosis model (Sonoda et al., 2020).

Extracellular vesicles contain proteins, cellular plasma, and nucleotide bases, and especially microRNAs (miRNAs) (van Niel et al., 2018). miRNAs play critical roles in a variety of biological processes, including bone formation, resorption, remodeling, and bone cell differentiation (Jing et al., 2015; Kocijan et al., 2016). Previous work associated miR-29b with histomorphometric parameters of bone formation and microstructure parameters in idiopathic osteoporosis (Feichtinger et al., 2018). Furthermore, miR-29b was identified as a contributor for osteogenic differentiation of MSCs in several studies (Zhang et al., 2019; Xia et al., 2020). Moreover, a previous study revealed that BMSC-derived exosomal miR-29b could regulate aging-related insulin resistance (Su et al., 2019). However, there is limited knowledge about whether BMSCs use EVs to shuttle miR-29b and thus modulate their intrinsic ability of osteogenic differentiation. A Web-available bioinformatics analysis has revealed the miRNA reorganization sites between miR-29b and lysine demethylase 5A (KDM5A) (formerly known as retinoblastoma binding protein 2), which has a histone demethylase activity. Histone methylation is regulated by lysine (K)-specific demethylases (Kdms) (Pedersen and Helin, 2010), and KDM5A-mediated H3K4me3 demethylation has been shown to participate in the etiology of osteoporosis (Wang et al., 2016). KDM5A is enriched in the suppressor of cytokine signaling 1 (SOCS1) promoter region in resting natural killer cells, resulting in reduced H3K4me3 modification and altered chromatin reconfiguration (Zhao et al., 2016). Furthermore, it has been reported that SOCS1 enhances osteoblast differentiation (Wu et al., 2012), and that SOCS1 is an upstream regulator of nuclear factor (NF)-κB activation that can directly bind to NF-κB-p65, thus inhibiting NF-κB activation (Liu X. et al., 2016). Other research shows that NF-κB inhibits osteogenic differentiation of MSCs by promoting β-catenin degradation (Chang et al., 2013). As such, previous evidence implies a signaling axis involving miR-29b, KDM5A, SOCS1 promoter, and NF-κB in the pathway of osteogenic differentiation. In the study, we demonstrated that EV-encapsulated miR-29b-3p derived from BMSCs specifically targets the demethylase KDM5A, regulating H3K4me3 and H3K27ac at the promoter region of SOCS1, thus affecting its expression and the downstream NF-κB pathway to regulate osteogenic differentiation of BMSCs.

## Materials and Methods

### Sample Collection

Bone marrow specimens were derived from dissected femoral head tissues of 35 osteoporotic patients (osteoporosis group) and 20 non-osteoporotic patients (normal group) during total hip arthroplasty (THA). The comorbid presence of rheumatoid arthritis, cancer, and metabolic diseases were excluded in all 55 subjects. Human BMSCs (hBMSCs) were isolated by their adherence to plastic and maintained in phenol-free α-Minimum Essential Medium (MEM) (HyClone, Logan, UT, United States) that contains 10% fetal bovine serum (FBS, Gibco, Grand Island, NY, United States), 100 U/ml penicillin (HyClone), and 100 g/ml streptomycin (HyClone) in an incubator (5% CO_2_, 37°C). Osteogenic induction experiments of hBMSCs were performed in the MEM with additional 50 g/ml of ascorbic acid (Sigma-Aldrich, St. Louis, MO, United States), 0.1 mg/ml dexamethasone (Sigma-Aldrich, St. Louis, MO, United States), and 10 mM glycerophosphate (Sigma-Aldrich, St. Louis, MO, United States).

### Cell Culture

hFOB1.19 cells were purchased from American Type Culture Collection (ATCC) and cultured in α-MEM medium containing 10% FBS in an incubator (5% CO_2_, 37°C). Passage was performed at a 1:4 ratio upon reaching 80–90% confluence. The cells at passage 3 were used for the experiments.

### Isolation of Extracellular Vesicles

The α-MEM (Gibco, Carlsbad, CA, United States) containing 20% FBS (Gibco, Carlsbad, CA, United States) was centrifuged at 200,000 × *g* for 18 h to deplete EVs, in which hFOB1.19 cells were cultured. hFOB1.19 cells were washed three times with phosphate buffered saline (PBS) and cultured in serum-free medium for 24–48 h. EVs were isolated from the supernatant as previously described (Li et al., 2017).

The EVs were fixed with 1% glutaraldehyde, placed on formvar-carbon-coated transmission electron microscopy (TEM) grids, and then stained with 1% phosphotungstic acid. The samples were visualized by JEM-2100 TEM (JEOL, Tokyo, Japan). The light sheet microscopy (LSM) images were recorded with the PARTICLEMEIRIX system. The EVs were analyzed with NanoSight tracking analysis (NTA) using the NanoSight NS300 system (Malvern Instruments, Malvern, United Kingdom) to record the Brownian motion of EVs using the Stokes–Einstein equation to calculate particle size distribution. The identified EVs were further confirmed by immunoblotting for markers of CD63 (1:2,000, ab216130; Abcam, United Kingdom), TSG101 (1:10,000, ab125011; Abcam), and Syntenin1 (1:100,000, ab133267; Abcam).

### Ovariectomized Osteoporosis Mouse Model

Twenty-five healthy C57BL/6J female mice, aged 12 weeks, were randomly assigned to five groups. The mice underwent either dorsal ovariectomy or sham operation (sham) under anesthesia with intraperitoneal (i.p.) 1% pentobarbital sodium (Liu Q. et al., 2016). agomiR-NC, agomiR-miR-29b-3p, or agomiR-miR-29b-3p + si-SOCS1 (all at a dose of 7 mg/kg body weight) was administered *via* i.p. injection into the ovariectomized (OVX) mice on the first 3 days of the first, third, and fifth weeks. Meanwhile, 0.2 ml PBS was injected into the sham-operated mice and OVX mice. All mice were subjected to micro-CT scanning before euthanasia.

### Real-Time Quantitative Polymerase Chain Reaction

TRIzol reagents were used to extract total RNA. The total RNA reverse transcription to cDNA was performed with the kit (K1622, Yaanda Biotechnology Ltd., Beijing, China). The cDNA was used for the subsequent real-time quantitative polymerase chain reaction (RT-qPCR) on a ViiATM 7 real-time PCR system (Daangene Ltd., Guangzhou, China). The expression level of genes relative to glyceraldehyde-3-phosphate dehydrogenase (GAPDH) or U6 was determined using the 2^–ΔΔCt^ method. The primers used are shown in Table 1.

**TABLE 1:** Primer sequences.

	Sequences (5′–3′)	
miR-29b-3p	F: TGCGGTAGCACCATTTGAAAT	R: CCAGTGCAGGGTCCGAGGT
KDM5A	F: GGTGTATCCGCAGAAATGG	R: TAGGAAGGGAGGAGGTGGT
SOCS1	F: AGCTCCTTCCCCTTCCAGATT	R: CCACATGGTTCCAGGCAAGTA
GAPDH	F: GAAGGTGAAGGTCGGAGTC	R: GAAGATGGTGATGGGATTTC
U6	F: CTGGTAGGGTGCTCGCTT	R: CGGCAGCAACTGGTGTCG

*GAPDH, glyceraldehyde-3-phosphate dehydrogenase; KDM5A, lysine demethylase 5A; NF-κB, nuclear factor-κB; SOCS1, suppressor of cytokine signaling 1.*

### Oil Red O Staining and Alizarine Red Staining

As previously reported (Fang et al., 2019), osteogenic differentiation in hFOB1.19 cells was analyzed by Oil red O staining and alizarine red staining.

### Western Blot Analysis

Protein samples were separated by sodium dodecyl sulfate–polyacrylamide gel electrophoresis (SDS-PAGE) and then transferred to polyvinylidene fluoride (PVDF) membranes. The membranes were blocked with 5% bovine serum albumin (BSA) at room temperature for 1 h, followed by incubation with the primary antibodies overnight at 4°C and subsequently with secondary antibodies at room temperature for 1.5 h. A standard enhanced chemiluminescence reagent kit (NCI4106, Pierce, Rockford, IL, United States) was used to detect the immunoreactive proteins. The used antibodies were as follows: anti-KDM5A (1:1,000, ab70892; Abcam), anti-SOCS1 (1:1,000, ab3691; Abcam), anti-p65 (1:1,000), anti-p-p65 (1:1,000, A7169; Assay Biotech, United States), anti-β-actin (1:5,000, ab75186), and horseradish peroxidase (HRP)-labeled immunoglobulin G (IgG) (1:20,000, ab205718).

### Chromatin Immunoprecipitation Assay

Lysine demethylase 5A enrichment at the promoter region of SOCS1 gene was evaluated by chromatin immunoprecipitation (ChIP) assays using an EZ ChIP Chromatin Immunoprecipitation Kit (Millipore, Temecula, CA, United States). Primers for the promoter region of human SOCS1 gene were as follows: 5′-TCC AAG AAG GGT CGA GAT TG-3′, 5′-CCC GCT CTT TTG CTC TAC CT -3′ (Invitrogen, Shanghai, China) (Zhao et al., 2016).

### Immunofluorescence Staining

Bone marrow-derived mesenchymal stem cells were incubated with primary antibody against p65 (Abcam; ab16502, 1:500) at 4°C overnight. The primary antibody was detected by using Alexa Fluor 647-conjugated anti-rabbit IgG secondary antibody (Abcam; ab150075, 1:400). After the final wash, the nuclei were counterstained by 4′,6-diamidino-2-phenylindole (Sigma-Aldrich) for 5 min before imaging. Cells were visualized using a fluorescence microscope (IX73, Olympus, Japan).

### Luciferase Reporter Assay

The wild-type or mutant [UGGUGCU sequences in KDM5A 3′ untranslated region (3′UTR) binding region were mutated by random sequence] KDMA 3′UTR sequence was cloned to pmirGLO luciferase plasmid (Promega, Madison, WI, United States) *via Xho*I and *Bam*HI. The luciferase plasmids were delivered into HEK293T cells with miR-29b-3p mimic or negative control (NC) mimic. The cells were harvested at 48 h after transfection and lysed with lysis buffer. The luciferase activity was detected with the Dual-Luciferase^®^ Reporter Assay System kit (Promega, United States) on a Luminometer TD-20/20 plate reader (E5311, Promega, United States).

### Statistical Analysis

All data were expressed as mean ± standard deviation and analyzed by SPSS 21.0 software (IBM, NY, United States). Data between two groups were analyzed by unpaired Student’s *t*-test and by one-way ANOVA followed by Tukey’s multiple for more than two groups. Pearson correlation coefficient was used to analyze the correlation of the data of two groups. Statistical significance was considered when *p* < 0.05.

## Results

### Poorly Expressed miR-29b-3p in Bone Marrow-Derived Mesenchymal Stem Cell-Derived Extracellular Vesicles Sourced From Osteoporotic Patients

**FIGURE 1:**
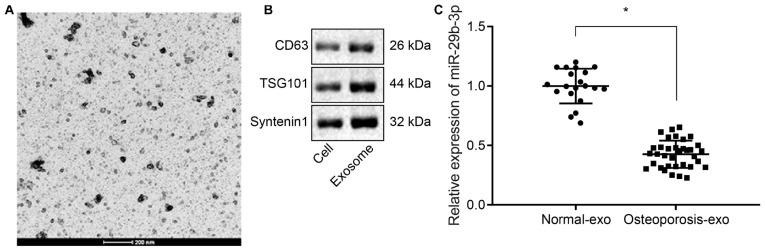
Low expression of miR-29b-3p in human bone marrow-derived mesenchymal stem cell (hBMSC)-derived extracellular vesicles (EVs) isolated from osteoporotic patients. **(A)** EVs were isolated from bone marrow samples of 35 osteoporotic patients (osteoporosis group) and 20 non-osteoporotic patients (normal group) during total hip arthroplasty (THA), and their morphology was observed by transmission electron microscopy (200 nm). **(B)** Western blot analyses of EV specific markers, CD63, TSG101, syntenin-1. **(C)** The expression of miR-29b-3p in hBMSC-derived EVs obtained from 35 osteoporotic patients (osteoporosis group) and 20 non-osteoporotic patients (normal group) was determined by real-time quantitative polymerase chain reaction (RT-qPCR). *p < 0.05 compared to normal.

Extracellular vesicles were isolated from hBMSCs from deserted femoral head tissues collected from osteoporotic patients and non-osteoporotic patients by ultracentrifugation, and their identity was confirmed by TEM and western blot analysis. The TEM examination revealed that the isolated EVs were round-shaped particles with a double-layered membrane structure (Figure 1A). Western blot analysis results showed that the EVs were positive for exosomal specific marker syntenin-1, TSG101, and CD63 (Figure 1B). RT-qPCR results revealed that the expression of miR-29b-3p was decreased in hBMSC-derived EVs of osteoporotic patients compared to non-osteoporotic patients (Figure 1C).

### Extracellular Vesicle-Encapsulated miR-29b-3p Released by Bone Marrow-Derived Mesenchymal Stem Cells Potentiated Osteogenic Differentiation by Specifically Inhibiting the Expression of KDM5A

**FIGURE 2:**
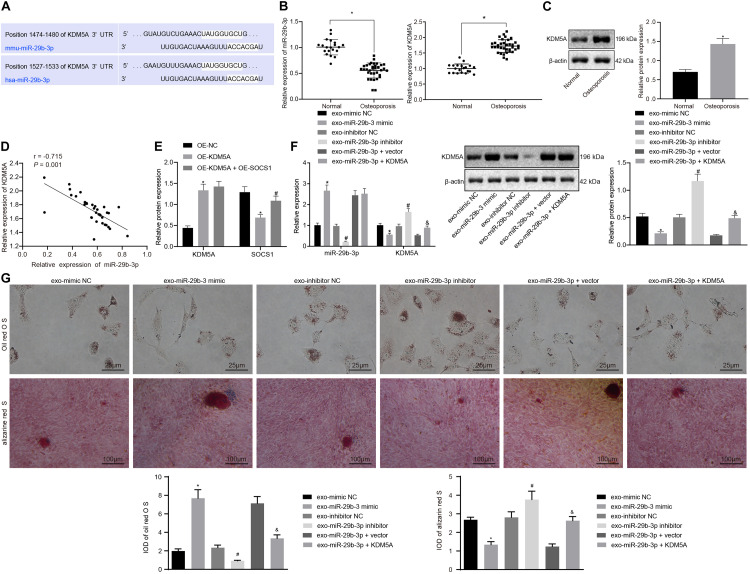
Extracellular vesicle (EV)-encapsulated miR-29b-3p-derived from bone marrow-derived mesenchymal stem cells (BMSCs) potentiated osteogenic differentiation by specifically inhibiting the expression of lysine demethylase 5A (KDM5A). **(A)** Bioinformatics prediction of the binding site of miR-29b-3p on KDM5A. **(B,C)** The expression of miR-29b-3p and KDM5A in the EVs of hBMSC isolated from osteoporotic patients or non-osteoporotic patients was detected by real-time quantitative polymerase chain reaction (RT-qPCR) **(B)** and western blot analysis **(C)**. *p < 0.05 compared to non-osteoporotic patients. **(D)** Pearson correlation analysis of miR-29b-3p and KDM5A expression levels in osteoporotic patients. **(E)** The interaction of miR-29b-3p with KDM5A was detected by dual-luciferase reporter assays. *p < 0.05 compared to NC mimic + wild-type (WT) KDM5A 3′ untranslated region (3′-UTR). **(F)** The expression of miR-29b-3p and KDM5A after the overexpression or knockdown of miR-29b-3p was analyzed by RT-qPCR and western blot analysis. **(G)** Osteogenic differentiation was analyzed by Oil red O staining and alizarine red staining (undifferentiated cells were indicated by the red colored lipid droplets, 400×). *p < 0.05 compared to the EV-mimic NC group, ^#^*p* < 0.05 compared to the EV inhibitor NC group, ^&^*p* < 0.05 compared to the EV-miR-29b-3p + vector group. The results represent three independent experiments.

To study the putative mechanisms of the miR-39b-3p-mediated regulation of KDM5A, the bioinformatics predictions using starBase software^1^ revealed the miR-29b-3p binding sites in the KDM5A mRNA 3′UTR (Figure 2A). RT-qPCR and western blot analysis revealed reduced expression of miR-29b-3p but increased expression of KDM5A in hBMSCs derived from osteoporotic patients compared to those in non-osteoporotic patients (Figures 2B,C). Furthermore, the expression of miR-29b-3p was negatively correlated with KDM5A in osteoporosis samples (Figure 2D). Subsequently, dual-luciferase reporter assays were performed to determine the interaction between miR-29b-3p and KDM5A. As shown in Figure 2E, a significant decrease in relative luciferase activity was noted when wild-type KDM5A was co-transfected with miR-29b-3p mimic (WT + miR-29b-3p vs. WT + control). However, the relative luciferase activity was comparable when mutant KDM5A was co-transfected with control plasmid (mutant + control) or miR-29b-3p mimic (mutant + miR-29b-3p mimic). These results indicate that miR-29b-3p specifically targeted KDM5A.

We then transfected hBMSCs with miR-29b-3p mimic and miR-29b-3p inhibitor and then isolated EVs. The isolated EVs were cocultured with osteoblasts with or without KDM5A overexpression. It was revealed that the miR-29b-3p expression was elevated, while expression of KDM5A was declined in the EV-miR-29b-3p mimic group compared to the EV-mimic NC group. In contrast, the EV-miR-29b-3p inhibitor group showed a decreased miR-29b-3p level and increased expression of KDM5A compared to the EV inhibitor NC group (Figure 2F). When compared to the EV-miR-29b-3p + vector co-transfection, co-transfection of miR-29b-3p and KDM5A significantly elevated the expression of KDM5A (Figure 2F). We subsequently performed Oil red O staining and alizarine red staining and found that the osteoblasts had enhanced differentiation capacity upon EV-miR-29b-3p mimic treatment but reduced differentiation capacity upon EV-miR-29b-3p inhibitor treatment (Figure 2G). Moreover, co-transfection of miR-29b-3p and KDM5A also reduced osteoblast differentiation capacity (Figure 2G).

### KDM5A Inhibited Osteogenic Differentiation by the Regulation of H3k4me3 and H3k27ac at the Suppressor of Cytokine Signaling 1 Promoter

**FIGURE 3:**
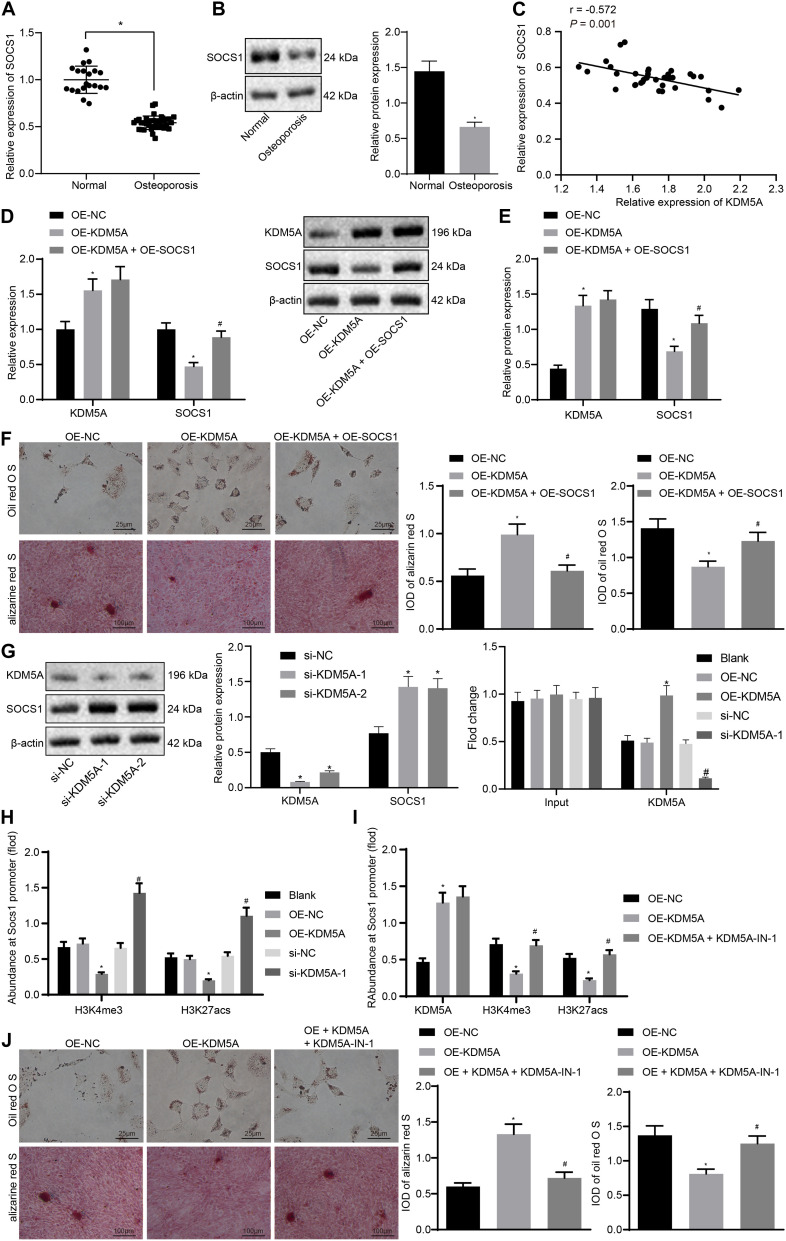
Lysine demethylase 5A (KDM5A) inhibited osteogenic differentiation by the regulation of H3K4me3 and H3K27ac of suppressor of cytokine signaling 1 (SOCS1). **(A,B)** The mRNA and protein levels of SOCS1 after overexpression or knockdown of KDM5A were detected by real-time quantitative polymerase chain reaction (RT-qPCR) and western blot analysis. *p < 0.05 compared to control. **(C)** Correlation analysis of KDM5A and SOCS1 in osteoporotic patients. **(D,E)** mRNA and protein levels of KDM5A and SOCS1 after overexpression or knockdown of KDM5A and SOCS1 were detected by RT-qPCR **(D)** and western blot analysis **(E)**. **(F)** Osteogenic differentiation was analyzed by Oil red O staining and alizarine red staining (400×). *p < 0.05 compared to the OE-NC group, ^#^*p* < 0.05 compared to the OE-KDM5A group. **(G)** The protein level of KDM5A and SOCS1 after transfection of different plasmids was detected by western blot analysis (left), and the binding of KDM5A in the promoter region of SOCS1 gene after transfection was detected by chromatin immunoprecipitation (ChIP) (right). *p < 0.05 compared to the OE-NC group, ^#^*p* < 0.05 compared to the si-NC group. **(H)** After overexpression or knockdown of KDM5A, the promoter of SOCS1 was immunoprecipitated by antibodies against KDM5A, H3K4me3, and K3K27acs and analyzed by RT-qPCR. *p < 0.05 compared to the si-NC group. **(I)** After treatment with the protein demethylase inhibitor, the promoter of SOCS1 was immunoprecipitated by antibodies against KDM5A, H3K4me3, and K3K27acs and analyzed by RT-qPCR. **(J)** Osteogenic differentiation was analyzed by Oil red O staining and alizarine red staining (400×). *p < 0.05 compared to the OE-NC group, ^#^*p* < 0.05 compared to the OE-KDM5A group.

To investigate whether KDM5A inhibits the expression of SOCS1 in hBMSCs, KDM5A, and SOCS1 expression in hBMSCs isolated from osteoporotic patients and non-osteoporotic patients was determined. As shown in Figures 3A,B, the expression of SOCS1 was decreased in osteoporotic patients compared to that of non-osteoporotic patients. Moreover, the expression of SOCS1 was negatively correlated with KDM5A (Figure 3C).

To further investigate the relationship between KDM5A and SOCS1 during osteogenic differentiation, we then transfected overexpressed KDM5A or SOCS1 in hBMSCs. The results of RT-qPCR and western blot analysis displayed OE-KDM5A led to increased KDM5A. However, the expression of SOCS1 was decreased in the OE-KDM5A group compared to that of the OE-NC group (Figures 3D,E). Alizarine red S staining and Oil red O staining results demonstrated that the differentiation potential of hBMSCs transfected with overexpressed KDM5A was markedly inhibited, which could be rescued by overexpressed SOCS1 (Figure 3F).

We observed reduced expression of SOCS1 upon KDM5A knockdown by si-KDM5A-1 and si-KDM5A-2 (Figure 3G, left) and found less SOCS1 expression upon treatment with si-KDM5A-1. We then detected the binding of KDM5A to the promoter region of SOCS1 by ChIP assay (Figure 3G, right). The binding of KDM5A to the promoter region in the OE-NC or si-NC group was similar to that of the blank group (*p* > 0.05). OE-KDM5A transfection resulted in more KDM5A enrichment at the promoter region of SOCS1, whereas si-KDM5A-1 and si-KDM5A-2 transfection yielded an opposite trend. Taken together, these results indicated that KDM5A bound to the promoter region and regulated the expression of SOCS1. After knockdown of KDM5A in the osteoblasts, the binding of H3K4me3 and H3K7acs on the promoter region of SOCS1 was increased dramatically (Figure 3H).

We then detected the effect of treatment with the protein demethylase inhibitor KDM5A-IN-1 on the expression of KDM5A in osteoblasts with KDM5A overexpression. The KDM5A expression was increased in the OE-KDM5A group, while KDM5A-IN-1 did not affect KDM5A expression compared to that in the OE-NC group (Figure 3I). The level of H3K4me3 and H3K27acs in the promoter region of SOCS1 in the OE-KDM5A group was increased compared to that of the OE-NC group, but KDM5A-IN-1 treatment dramatically decreased H3K4me3 and H3K27acs levels compared to those in the OE-NC group (Figure 3I). Overexpression of KDM5A inhibited the differentiation of BMSCs, while KDM5A-IN-1 enhanced the differentiation of BMSCs (Figure 3J).

### Suppressor of Cytokine Signaling 1 Potentiated Osteogenic Differentiation by Inhibiting the Nuclear Factor-κB Pathway

**FIGURE 4:**
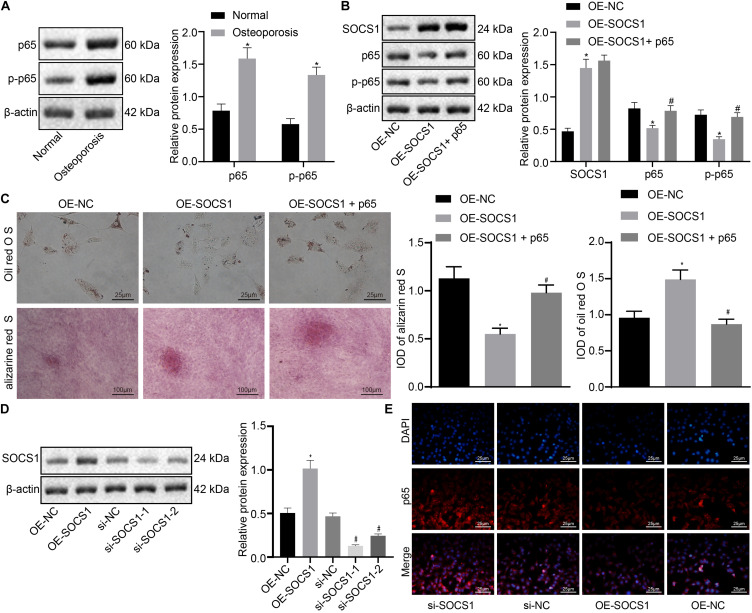
Suppressor of cytokine signaling 1 (SOCS1) potentiated osteogenic differentiation by inhibiting the nuclear factor (NF)-κB pathway. **(A)** The expression of p65 and p-p65 in the human bone marrow-derived mesenchymal stem cells (hBMSCs) sourced from osteoporotic patients and non-osteoporotic patients. *p < 0.05 compared to non-osteoporotic patients. **(B)** The expression of SOCS1 and p65 was detected by western blot analysis. **(C)** Osteogenic differentiation was analyzed by Oil red O staining and alizarine red staining (400×), *p < 0.05 compared to the OE-NC group, ^#^*p* < 0.05 compared to the OE-SOCS1 group. **(D)** The expression of SOCS1 was detected in SOCS1-overexpressing or SOCS1 knockdown cells by western blot analysis. *p < 0.05 compared to the OE-NC group, ^#^*p* < 0.05 compared to the si-NC group. **(E)** Translocation of p65 to the nucleus was detected in SOCS1-overexpressing or SOCS1 knockdown cells by immunofluorescence staining (400×).

To confirm whether SOCS1 potentiates osteogenic differentiation by blocking the NF-κB pathway, the NF-κB [p65 and phosphorylated p65 (p-p65)] in the hBMSCs of osteoporotic patients and non-osteoporotic patients was detected. As shown in Figure 4A, the expression of NF-κB was markedly increased in hBMSCs sourced from osteoporotic patients.

To investigate the effect of SOCS1 and NF-κB on osteogenic differentiation, we constructed the overexpression of SOCS1/p65 in osteoblasts. The expression of SOCS1 was demonstrably increased while that of NF-κB (p65, p-p65) was decreased in the OE-SOCS1 group compared to those in the OE-NC group. However, the expression of SOCS1 was comparable while that of NF-κB was increased markedly in cells co-transfected with both SOCS1 and p65 (OE-SOCS1 + p65) compared to those in cells transfected with SOCS-1 only (Figure 4B). Osteogenic differentiation experiments showed that the differentiation capacity of cells in the OE-SOCS1 group was markedly potentiated compared to that of the OE-NC group. Consistent with this result, cells in the OE-SOCS1 + p65 group showed decreased differentiation capacity compared to that in the OE-SOCS1 group (Figure 4C). To confirm these results, we also produced knockdown of SOCS1 and detected the translocation of p65 to the nucleus transfected in osteoblasts. The expression of SOCS1 was markedly reduced both in the si-SOCS1-1 and si-SOCS1-2 groups. We saw more potent knockdown in the si-SOCS1-1 group and consequently used if for subsequent experiments (Figure 4D). Moreover, immunofluorescence staining demonstrated that p65 translocation into the nucleus was inhibited in SOCS1-overexpressing cells (Figure 4E).

### Extracellular Vesicle-Encapsulated miR-29b-3p Potentiated Osteogenic Differentiation Through Blocking Suppressor of Cytokine Signaling 1/Nuclear Factor-κB Pathway by Inhibiting KDM5A

**FIGURE 5:**
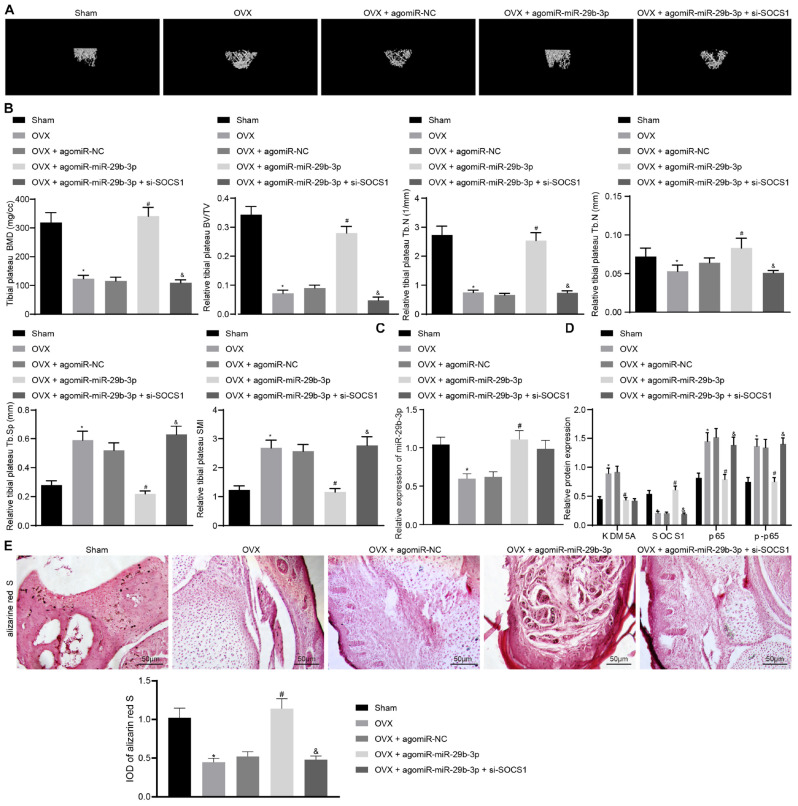
Extracellular vesicle (EV)-encapsulated miR-29b-3p potentiated osteogenic differentiation through blocking the suppressor of cytokine signaling 1 (SOCS1)/nuclear factor (NF)-κB pathway by inhibiting lysine demethylase 5A (KDM5A). **(A)** Mice femur image was acquired by micro-CT scanning. **(B)** Quantitative analysis of bone mineral density (BMD), trabecular bone volume/tissue volume (TBV/TV), trabecular number (Tb.N), trabecular separation (Tb.Sp), and structural model index (SMI) of the mouse femur. **(C)** miR-29b-3p level in the EVs was determined by real-time quantitative polymerase chain reaction (RT-qPCR). **(D)** The expression of KDM5A, SOCS1, and NF-κB (p65, p-p65) in mouse bone tissues was detected by western blot analysis. **(E)** Osteogenic differentiation in mouse bone tissues was analyzed by alizarine red staining (200×). *p < 0.05 compared to the sham group, ^#^*p* < 0.05 compared to the OVX + agomiR-NC group, ^&^*p* < 0.05 compared to the OVX + agomiR-miR-29b-3p group.

Micro-CT results demonstrated that the BMD, trabecular bone volume/tissue volume (TBV/TV), trabecular number (Tb.N), and trabecular thickness (Tb.Th) were decreased significantly. Meanwhile, the trabecular separation (Tb.Sp) and structural model index (SMI) were increased in osteoporosis mice compared to those in sham-operated mice (Figures 5A,B). These results strongly confirmed that osteoporosis was successfully induced in OVX mice.

Nanocomplex/aptamer-agomiR-29b-3p was injected into the abdominal cavity of young mice (Su et al., 2019), and micro-CT was then performed, which demonstrated that the BMD, TBV/TV, and Tb.N thickness were increased significantly while the Tb.Sp and SMI were markedly thinned in OVX mice treated with agomiR-29b-3p injection (Figures 5A,B). This indicated that osteoporosis was relieved by agomiR-29b-3p. By contrast, the BMD, TBV/TV, Tb.N, and Tb.Th thickness were decreased significantly while the Tb.Sp and SMI thickness were increased markedly in OVX mice treated with agomiR-miR-29b-3p and si-SOCS1 injection compared to those in OVX mice with only agomiR-miR-29b-3p injection (Figures 5A,B), thus confirming that these mice had osteoporosis.

Extracellular vesicles were isolated from the above mice. We found that OVX mice with agomiR-miR-29b-3p injection displayed an elevated miR-29b-3p level (Figure 5C). We also detected the expression of KDM5A, SOCS1, and NF-κB (p65, p-p65) in these mice and found the expression of KDM5A, p65, and p-p65 to be reduced, whereas the expression of SOCS1 was increased in OVX mice with agomiR-miR-29b-3p injection. Compared to OVX mice with agomiR-miR-29b-3p injection, OVX mice with both agomiR-miR-29b-3p and si-SOCS1 injection displayed reduced expression of SOCS1 and elevated expression of p65 and p-p65 (Figure 5D). Alizarine red staining demonstrated that OVX mice with agomiR-miR-29b-3p injection showed increased osteogenic differentiation capacity, while OVX mice with both agomiR-miR-29b-3p and si-SOCS1 injection had reduced osteogenic differentiation capacity when compared to OVX mice with agomiR-miR-29b-3p injection (Figure 5E).

## Discussion

In this study, we demonstrated that miR-29b-3p was significantly downregulated in BMSC-derived EVs of osteoporotic patients. The decreased expression of miR-29b-3p subsequently resulted in upregulation of KDM5A, which is a demethylase regulating the expression of a variety of genes by its epigenetic modification of the gene promoters. Increased expression of KDM5A inhibited the expression of SOCS1 by demethylation of H3K4me3 and H3K27ac in the promoter region of SOCS1. This event resulted in the loss of the inhibitory effect of SOCS1 on the expression of NF-κB p65. The expression and translocation of p65 to the nucleus in turn inhibited the osteogenic differentiation of BMSCs, leading to osteoporosis. Thus, decreased expression of miR-29b-3p in the BMSC is one of the causes of osteoporosis.

Bone metabolic disease, for example, osteoporosis, is characterized by the imbalance of osteoblasts and osteoclasts (Ensrud and Crandall, 2017). Recent efforts have been made to characterize osteoclast–osteoblast coupling factors in humans, aiming to reveal a link between bone growth and energy metabolism (Weivoda et al., 2020). MSCs are normally able to produce corresponding osteoclasts to offset the bone absorption through regulation of osteoclasts (Aghebati-Maleki et al., 2019). Therefore, studying the osteogenic differentiation of MSC is a key point for exploring the osteoporosis pathogenesis pathway. The pro-osteogenic effect of BMSCs is enhanced by their paracrine action, such as delivery of EVs (Chu et al., 2019). Recent studies have indicated that BMSC differentiation into osteoblasts is regulated by transfer *via* EVs of protein cargos or miRNAs such as miR-31a-5p (Xu et al., 2018) and miR-21 (Jiang et al., 2018). We placed our attention on the mediation of miR-29b in the control of the ability of BMSCs to differentiate into osteoblasts. As expected, we observed that the release of EV-encapsulated miR-29b-3p from BMSCs enhanced osteogenic differentiation. Several studies have presented similar results. For example, miR-29b was associated with histomorphometric parameters of bone formation and microstructure parameters in idiopathic osteoporosis (Feichtinger et al., 2018). miR-29b has been found in several studies to be a contributor for osteogenic differentiation of MSCs (Zhang et al., 2019; Xia et al., 2020). The miR-29 family contains miR-29a, miR-29b, miR-29c, which differ only in two or three bases (Chen et al., 2017). It was reported that the expression of miR-29 family members is elevated in cartilage during osteoarthritis (OA), which was suppressed by SRY-box transcription factor 9 (SOX9) in chondrocytes (Le et al., 2016). Decreased expression of miR-29 family members has been shown to be important for chondrogenic differentiation of MSCs by targeting Forkhead box class O 3A (FOXO3A) (Guerit et al., 2014). miR-29b-3p, a member of the miR-29 family, was recently shown to regulate extracellular matrix formation through the regulation of collagen type I expression (Li et al., 2009). There are also publications reporting that miR-29b-3p decreases the expression of many genes involved in the regulation of osteoblast formation, such as histone deacetylase 4 (HDAC4), transforming growth factor-β3 (TGFb3), activin receptor type 2A (ACVR2A), beta-catenin-interacting protein 1 (CTNNBIP1), and dual-specificity protein phosphatase 2 (DUSP2) (Franceschetti et al., 2013). Whether these genes affect miR-29b-3p regulation of osteogenic differentiation merits further investigations.

In this study, we sought to determine whether KDM5A serves as the downstream mechanism of miR-29b in the control of the ability of BMSCs to differentiate into osteoblasts. miR-29b-3p targeting of KDM5A was confirmed by dual-luciferase reporter assay. KDMs have been widely reported to be involved in the regulation of MSC lineage specification (Cloos et al., 2008). KDM4B, the demethylase K9 of histone H3, was activated by bone morphogenetic protein (Pedersen and Helin, 2010). KDM4B is required for osteogenic differentiation of MSCs by removing H3K9me3 at the Dlx5 promoter region (Ye et al., 2018). KDM4B also has a critical role in TGFb-mediated chondrogenic differentiation of MSCs *via* recruitment of mothers against decapentaplegic homolog 3 (SMAD3) and Sox9 (Lee et al., 2016). Similarly, knockdown of KDM6A inhibited Runx2 expression by promotion of H3K27me3 on the promoter regions and thus suppressed osteogenic differentiation (Wang et al., 2016). In this study, KDM5A led to demethylation of H3K4me3 in the SOCS1 promoter region and reduce osteogenic differentiation of BMSCs. Furthermore, knockdown of KDM5A elevated the level of H3K4me3 and potentiated osteogenic differentiation of BMSCs, whereas overexpression of KDM5A reduced the level of H3K4me3 and blocked osteogenic differentiation. The effect of KDM5A on the regulation of H3K4me3 depends on its demethylase activity, as shown by the present results that treatment with a demethylase inhibitor also upregulated the level of H3K4me3 and osteogenic differentiation of BMSCs. KDM5A is enriched at the SOCS1 promoter region in resting natural killer cells, leading to a decline in H3K4me3 modification and suppressed chromatin configuration (Zhao et al., 2016), whereas SOCS1 enhances osteoblast differentiation (Wu et al., 2012). As shown in our study, miR-29b-mediated inhibition of KDM5A leads to promotion of SOCS1, thus enhancing osteoblast differentiation. SOCS1 suppresses the NF-κB-dependent expression of inflammatory genes and serve as a causative factor of lipopolysaccharide (LPS) -induced inflammation in macrophages (Strebovsky et al., 2011). SOCS1 directly binds to the p65 subunit of NF-κB and facilitates ubiquitination of the subunit, resulting in the degradation of p65 (Ryo et al., 2003). Consistent with these results, we found that expression of SOCS1 negatively correlates with that of p65 in BMSCs. A reduced level of SOCS1 results in increased expression and translocation of p65 to the nucleus, while overexpression of SOCS1 leads to decreased expression and translocation of p65 to the nucleus. The osteogenic differentiation, depending on the level of NF-κB, is affected accordingly.

## Conclusion

**FIGURE 6:**
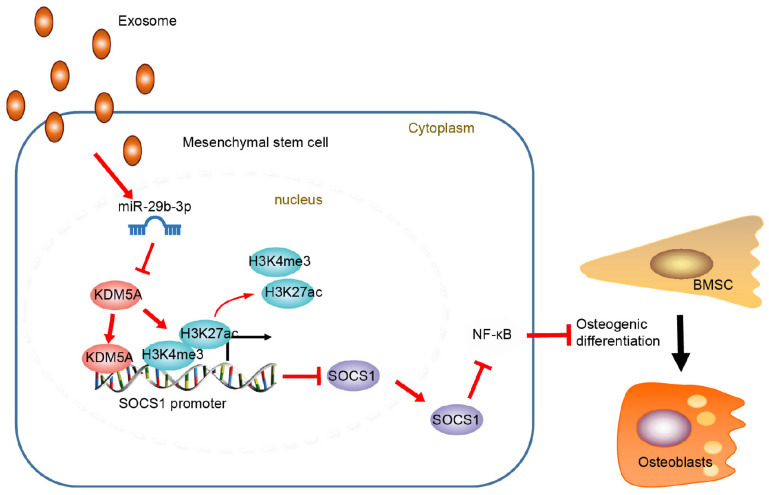
Schematic map displayed the underlying mechanism concerning extracellular vesicle (EV)-encapsulated miR-29b-3p-derived from bone marrow-derived mesenchymal stem cells (BMSCs) in osteoporosis. EV-encapsulated miR-29b-3p-derived from BMSCs potentiated osteogenic differentiation through blocking the suppressor of cytokine signaling 1 (SOCS1)/nuclear factor (NF)-κB pathway by inhibiting lysine demethylase 5A (KDM5A).

Extracellular vesicle-encapsulated miR29b-3p promotes osteoblastogenesis both *in vitro* and *in vivo*, indicating that the reduced level of EV-encapsulated miR-29b-3p in osteoporotic patients is one of the major causes of this disease (Figure 6). On this basis, upregulation of EV-encapsulated miR-29b-3p could serve as an effective anabolic therapeutic strategy in osteoporotic patients. However, future investigations are warranted to confirm the role of miR29-3b in mediating BMSC differentiation in transgenic mice.

## Data Availability Statement

The original contributions presented in the study are included in the article/supplementary material, further inquiries can be directed to the corresponding author/s.

## Ethics Statement

The approval of experiments in this study was obtained from the Research Ethics Committee of the First Hospital of Lanzhou University. The written informed consent had been obtained from all patients provided specimens for this study.

## Author Contributions

XZ and YW designed the study. WW and TZ were involved in the data collection. HZ and PQ performed the statistical analysis and preparation of figures. XH drafted the manuscript. All authors read and approved the final manuscript.

## Conflict of Interest

The authors declare that the research was conducted in the absence of any commercial or financial relationships that could be construed as a potential conflict of interest.

## Footnotes

1 http://starbase.sysu.edu.cn
